# Hidden hunger? Experiences of food insecurity amongst Pakistani and white British women

**DOI:** 10.1108/BFJ-06-2018-0342

**Published:** 2018-11-05

**Authors:** Maddy Power, Neil Small, Bob Doherty, Kate E. Pickett

**Affiliations:** 1 University of York, York, UK; 2 University of Bradford, Bradford, UK

**Keywords:** Pakistani, Ethnic groups, Lived experience, Shame, Food banks, Food poverty

## Abstract

**Purpose:**

Foodbank use in the UK is rising but, despite high levels of poverty, Pakistani women are less likely to use food banks than white British women. The purpose of this paper is to understand the lived experience of food in the context of poverty amongst Pakistani and white British women in Bradford, including perspectives on food aid.

**Design/methodology/approach:**

A total of 16 Pakistani and white British women, recruited through community initiatives, participated in three focus groups (one interview was also held as a consequence of recruitment difficulties). Each group met for two hours aided by a moderator and professional interpreter. The transcripts were analysed thematically using a three-stage process.

**Findings:**

Women in low-income households employed dual strategies to reconcile caring responsibilities and financial obligations: the first sought to make ends meet within household income; the second looked to outside sources of support. There was a reported near absence of food insecurity amongst Pakistani women which could be attributed to support from social/familial networks, resource management within the household, and cultural and religious frameworks. A minority of participants and no Pakistani respondents accessed charitable food aid. There were three reasons for the non-use of food aid: it was not required because of resource management strategies within the household and assistance from familial/social networks; it was avoided out of shame; and knowledge about its existence was poor.

**Originality/value:**

This case study is the first examination of varying experiences of food insecurity amongst UK white British and Pakistani women. Whilst the sample size is small, it presents new evidence on perceptions of food insecurity amongst Pakistani households and on why households of varying ethnicities do not use food aid.

## Introduction

This paper explores the lived experience of food in the context of poverty amongst Pakistani and white British women living in Bradford. It aims to present their perspectives on and experiences of food insecurity and charitable food aid, with a particular focus on ethnic differences.

In 2017, the Food Standards Agency (FSA, 2017) reported that 13 per cent of UK adults were marginally food secure and 8 per cent had low or very low food security. Food insecurity, the “limited or uncertain availability of nutritionally adequate and safe foods or limited or uncertain ability to acquire acceptable foods in socially acceptable ways” (Anderson, 1990, pp. 1575-1576), was found to disproportionately affect people living on low incomes and younger people. However, consistent with international evidence (Knowles *et al.*, 2015), households with young children were also at greater risk of food insecurity than the general population: respondents in households with children aged under 16 were more likely to report having made a change to their buying and eating arrangements for financial reasons than respondents in adults-only households (58 per cent compared with 37 per cent). Despite a considerable body of international evidence suggesting that children within food insecure households are protected from the more serious effects of food insecurity, i.e. hunger, qualitative research within the UK indicates that, amongst the most economically deprived food insecure families, parents may not be able to protect their children from the sharp impact of food insecurity (Harvey, 2016).

Inter- and intra-household food insecurity is highly gendered. Women in low-income households are at particular risk of food insecurity and households with children headed by single women are more likely to be food insecure than other household types, independent of socio-demographic characteristics (Alaimo *et al.*, 1998). Within the household, gender inequalities in food access and consumption may result from women’s actual or perceived role in the family as procurer of food and carer of children (Collins, 2009). Indeed, adult women in food insecure households have lower intakes of nutrients than other household members, including adult men (Rose, 1999), suggesting women may make choices that disproportionately adversely affect them as they seek to protect children and privilege men in the household.

### Household responses to food insecurity

Food insecure households reportedly adopt variegated “coping” techniques in proportion to their level of vulnerability. Food insecure households may reduce the quality and/or quantity of food consumed (Pfeiffer *et al.*, 2011), adopt meticulous budgeting strategies (Huisken *et al.*, 2017) and draw upon credit and loans (Perry *et al.*, 2014). While social and familial networks may be used for social, emotional and nutritional support (Pfeiffer *et al.*, 2011), the tendency or ability to seek support from social networks varies by demography. For instance, some parents describe reliance on others as “stressful and often threatening” (Ahluwalia *et al.*, 1998, p. 599), while African-American respondents may be more likely than other ethnic groups to depend upon formal support systems due to high poverty amongst their own social networks (Ahluwalia *et al.*, 1998).

Charitable food aid, including food banks, may be accessed as a “pragmatic” or “last resort” response to food insecurity in the context of an acute income crisis (Lambie-Mumford and Dowler, 2014), such as the ineffective operation of financial support from a social security system, a sudden loss of earnings or a change in family circumstances (Perry *et al.*, 2014). The discrepancy between national-level statistics on foodbank use and FSA data on UK food insecurity – in 2016–2017, the Trussell Trust network of foodbanks distributed 1,182,954 food parcels, less than would be expected given that roughly 4m people have low/very low food security (FSA, 2017) – underscores the extent to which accessing food aid may be a “last resort”, avoided entirely or (geographically) inaccessible. While there are many independent (non-Trussell Trust) food banks and informal sources of food aid, such as lunch clubs, the number of people receiving food from these organisations has not been systematically documented (Purdam *et al.*, 2015).

This “last resort” aspect suggests a considerable reluctance to seek food aid even in contexts of considerable need, a situation at odds with political and, at times, public rhetoric of people too easily resorting to aid. Indeed, it is well established that, for many, accessing food aid can be a stigmatising experience. Receiving food assistance may force an individual to abandon both embodied dispositions towards food and norms about obtaining food (van der Horst *et al.*, 2014), whilst placing them in an interaction of charitable giving which damages self-esteem (van der Horst *et al.*, 2014). The majority of contemporary research on the lived experience of food insecurity in the UK samples participants via food banks (predominantly Trussell Trust foodbanks), arguably rendering much of the literature restricted to an investigation of food insecurity amongst a specific population (Power, Doherty, Small, Teasdale and Pickett, 2017). There is an urgent need to understand why people who experience food insecurity in the UK do not use food aid and to discuss the process of using a food bank with people who access this support but are not recruited, for research purposes, through the food bank itself.

### Similarities and variations between Pakistani and white British households in food insecurity

International evidence identifies ethnic variations in the prevalence and experience of food insecurity. For instance, in the USA, Black and Mexican American households are more likely to be food insecure than the general population (Coleman-Jensen *et al.*, 2014). In the UK, despite greater deprivation and poorer health outcomes amongst Pakistanis in comparison with the white ethnic majority (Atkin, 2009), it is the latter, not the former, who are at higher risk of food insecurity (Power, Uphoff, Pickett, Stewart-Knox, Small and Doherty, 2018). Within the Global North there is no literature, to our knowledge, on varying responses to food insecurity within and between Pakistani and white British households. Nevertheless, the research on the role and importance of social networks within South Asian communities (Shaw and Charsley, 2006) does provide an insight into potentially different approaches to food in contexts of poverty amongst majority and minority ethnic groups.

Systems of reciprocity – in particular the sharing of food – operating amongst families in poverty within ethnic minority, including Pakistani, communities may function as an “informal security network”, mitigating the likelihood and impact of food insecurity (Fitchen, 1987, p. 319). Social prescriptions around food may not only shape the type of food purchased but limit the demography of socialisation to members of the ethnic minority group, which may, in turn, entrench reciprocation and thus also the security of food within the ethnic minority community (Vallianatos and Raine, 2008).

Beyond social networks, established systems of welfare provision within Pakistani Muslim communities may attenuate the extent of food insecurity. Zakat (compulsory almsgiving for Muslims), for instance, provides a practical and moral basis for welfare provision within Muslim societies (Dean and Khan, 1997). Whilst the giving of Zakat is an altruistic act, it also has a fundamental economic function: to seek a fair distribution and circulation of wealth (Ali, 1993, p. 88). In practice, Zakat tends to remain as a parallel or supplementary channel of revenue raising and distribution alongside the welfare state: the funds generated are employed partly to support international charitable programmes but also, more directly, to fund independent Islamic educational initiatives and promote welfare through individual grants to British Muslim families (Dean and Khan, 1997).

Notwithstanding the literature cited above, there is an apparent absence of UK evidence on the varying experiences within and between ethnic groups – in particular, white British and Pakistani people – on food consumption and food management in the home, in the context of poverty. Research into possible ethnic differences is all the more pressing given high levels of poverty amongst some South Asian communities in the UK (Atkin, 2009), including Bradford’s Pakistani community, and yet their apparent low use of charitable food aid for support (Power, Small, Doherty, Stewart-Knox and Pickett, 2017).

## Methodology

### Study site

The study was undertaken in Bradford, a city and metropolitan area in West Yorkshire with a population of over half a million (ONS, 2010). Bradford has the largest proportion of people of Pakistani ethnic origin of any local authority in England (20.4 per cent), which contributes to its large Muslim population (24.7 per cent). Bradford is the 19th most deprived local authority (out of 326) in England as measured by the Index of Multiple Deprivation (Gill, 2015) and scores substantially below country averages on most health indicators, even in comparison with other English cities marked by social and ethnic inequalities.

### Methods

Between July and November 2016, three focus groups and one interview were conducted with white British and Pakistani women in or at risk of food insecurity (*n*=16) living in Bradford. The inclusion of women only in the sample was motivated by two considerations: first, as described above, household food insecurity is a highly gendered experience with women at greater risk than men; and, second, this qualitative study emanated from quantitative work on ethnic differences in the prevalence and socio-demographics of food insecurity amongst Pakistani and white British women only; this qualitative study aimed to explore in depth the ethnic differences amongst women revealed by the quantitative analysis.

To mitigate potential recruitment difficulties and language and capacity restrictions, focus groups were arranged within pre-existing activity/community groups. With the assistance of Better Start Bradford (BSB) (a community initiative), existing group activities in Bradford in which it would be appropriate to hold focus groups were identified. Members of these groups were invited to participate in the study. The authors worked with BSB to ensure a diversity of groups and participants and, specifically, to include:

- white British and Pakistani women with dependent children;
- women who spoke only Urdu, women who were bilingual and women who spoke only English; and
- women living in severe deprivation, as well as those in low-income households[1].

**Figure 1:**
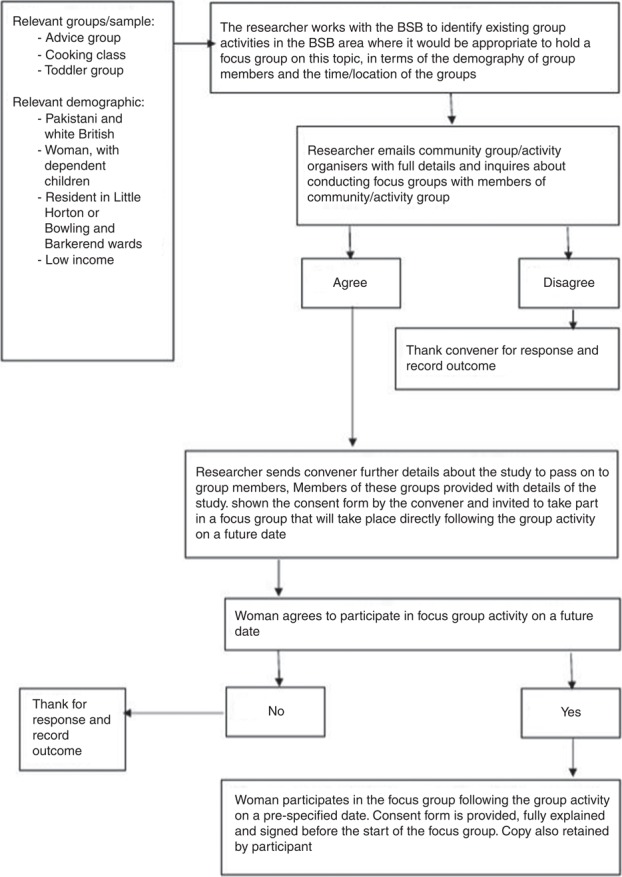
Recruitment process

Figure 1 sets out the recruitment process.

Three focus groups were conducted, and, as a consequence of recruitment difficulties, one interview was also held (recruitment difficulties included reluctance to consent to participation in a focus group and failure to attend the focus group at the pre-arranged time). The three focus groups and one interview were semi-structured, moderated by the first author and lasted between one and two hours.

The opening stage of the focus groups was conducted as an interview within a group (Morgan, 1997). Rather than presenting a question for whole group response, the moderator began by concentrating on a single participant, and subsequently requesting group members to respond. This approach aimed to involve all participants fully in the group and encourage each participant to give a meaningful response, with the goal of hearing from everyone (Morgan, 1997). As the focus group progressed the researcher acted as a “moderator” for the group (Wilkinson, 2004), rather than interviewer, posing the questions, maintaining the flow of the discussion and enabling members to participate fully.

Given that the aim of the study was to understand experiences as well as perceptions, the moderator at times directed the group discussions towards concrete and detailed accounts of the participants’ experiences. An emphasis on hearing about the participants’ experiences helped to counteract the movement towards generalities and generated a level of depth that drew the entire group into the discussion.

One focus group included participants with varying levels of English language ability. In this particular focus group, all participants were of Pakistani origin, however, while some (*n*=4) were bilingual (Urdu and English), others spoke only Urdu (*n*=3). Because of this, the focus group was conducted as two smaller conversation groups within the larger group, with Urdu speakers spoken with separately via a translator. Although there are significant limitations to dividing the group, it was preferred to excluding some members of the group on the basis of language or obstructing the flow of the conversation with a translator translating all dialogue – English and Urdu – to all participants.

The topic guide was discussed extensively with BSB, particularly staff who were members of Bradford’s Pakistani Muslim community, and with the convenors of the community groups in which the focus groups were to be held. It was piloted with two BSB staff members, one Pakistani Muslim and one secular white British. The focus groups were recorded on a Dictaphone and transcribed verbatim.

Full details of the 16 women in the sample are set out in Table I. To preserve the anonymity of participants identifying material is removed and direct quotes are presented with pseudonyms. There was a considerable range of socioeconomic status in the 16 participants; some women lived in households where no one worked and the only source of income was social security (*n*=3), others lived in households in which all adults could be categorised as in junior managerial, administrative or professional employment (*n*=3). The majority of participants (*n*=13) lived in a household where at least one adult was in paid employment. The ages of participants also varied, ranging from 18 to 48. Notably, half of the sample (*n*=8) were less than 25 years old. BSB, through which participants were recruited, focusses on early years’ intervention, working particularly with new parents, and this is reflected in the relatively young age of the sample. The Pakistani women in the sample were a less homogeneous group than the white British women; while all of the latter were born in the UK, three of the Pakistani women in the sample immigrated from Pakistani to Britain post-school.

Ethical consent was obtained from the University of York Department of Health Sciences Research Governance Committee (HSRGC) (Ref HSRGC/2015/121A). Given the vulnerability of some of the participants and the sensitive nature of the topic, ethical considerations were prominent in the design and conduct of the focus groups (and interview). The moderator aimed to ask participants about their personal experiences, however, the line of questioning was discontinued in situations where the participant appeared distressed. The moderator was also conscious of their position of power in their relationship with participants, in terms of both academic knowledge and their role in setting the agenda of the group, deciding the boundaries of time and indicating acceptable discussion points. The moderator attempted to address this power imbalance by foregrounding the right of the participant to withdraw at any time and providing the participant with considerable scope to determine the direction of the discussion. Participants were provided with full information about the study before agreeing to take part, and informed consent was attained before the start of each focus group/interview.

A three-stage analysis approach (Dwyer, 2002) was used to analyse the transcripts. Each transcript was initially summarised to understand the narrative. Thematic analysis was used; a coding frame was devised based upon common themes/sub-themes and, using Nvivo 10, this was applied to each transcript. Relevant text was indexed whenever a theme appeared. The appropriately indexed material was transferred to a grid with demographic details about the sample.

## Results

The nature, prevalence and – reported – concealment of food insecurity will be described, followed by a discussion on the lived experience of food insecurity, including consideration of ethnic differences. The section ends with an analysis of participants’ experiences and opinions, including avoidance of charitable food aid.

### The nature, prevalence and concealment of food insecurity

The reported experience of food insecurity varied starkly by ethnic group. Only one of the eight Pakistani women in the sample disclosed previous experiences of food shortages within the household compared with five of the eight white British women. The remaining seven Pakistani participants reported no issues with food insufficiency or financial barriers to food access. The possibility of food insecurity was assertively rejected: the price of food was described as “not a problem for us” or participants stated “we can afford whatever we need”. Three Pakistani participants, who spoke limited English and conversed only in Urdu, apparently struggled to understand a financial concept of food insecurity: questions about barriers to accessing food were answered in terms of language and knowledge obstacles to purchasing food only.

While all bar one Pakistani participant presented a narrative which implied food insecurity was avoided, discussion in the focus groups and interview intimated that food insecurity was indeed experienced by Pakistani households, but was concealed either from the wider South Asian community itself or from charitable and state support systems outside the community. Sabira, the only Pakistani woman to disclose food insecurity, described hiding her experience from members of the local community and from food banks, instead seeking support from her immediate family:

Even when life was very hard and money short, I would not go to a food bank because of shame and pride. You don’t want people to see you like that. There may be people you know there who will talk.

(Sabira)

The concealment of food insecurity was neither unique to the Pakistani community, nor was embarrassment stemming from food insecurity ethnically determined. White British participants similarly described the shame associated with food insecurity and their consequent concealment of food insufficiencies from the food bank, the community and, on occasion, the family. However, they also spoke more openly in the focus groups than their Pakistani counterparts about the lived experience of food insecurity.

### Food access

How and where food was purchased was discussed willingly by all participants. Descriptions of accessing food through normal channels – food delivery, supermarkets and local shops – could be conceptually divorced by most participants from financial restrictions to food access (food insecurity), alleviating sensitivities within the focus group associated with food insecurity. Obstacles to food access differed by ethnicity, or more specifically by migration status: participants who were Pakistani migrants and had settled in the UK after schooling in Pakistan (*n*=3) tended to foreground language and knowledge issues:

I find shopping overwhelmingly difficult because I don’t know where to buy food. So I rely on my family to get food for me.

(Ghada, translated from Urdu)

In halal food the meat is bled slowly so that the blood drains: the meat has less pain. But when this is rushed the meat is not good. It shows that the animal has suffered. I am worried about where I source my meat from.

(Faiza, translated from Urdu)

### Coping strategies

#### “Making ends meet” within the household

Cooking food from scratch, cooking in bulk and forming a meal from food available within the home were widely discussed by participants as strategies to “make ends meet”. Nevertheless, cooking – particularly from scratch or in bulk – was discussed more commonly and with greater fervour in the two focus groups containing those participants who were relatively more affluent. Amongst the most deprived participants, cooking using fresh ingredients and/or in bulk was subordinated to cooking not “healthy stuff” but “just what I could get really”.

Tight control of material resources within the household and keen attention to financial budgeting was a crucial – and cross-cultural – strategy to provide sufficient food. Weekly food shopping was carefully planned; household budgets were composed on a weekly, monthly and even yearly basis, and food was bought in bulk and at discounted rates:

Every week we do a shop. If something is on offer we get a few bits of whatever is on offer so we have always got something stored, and then this lasts a long time.

(Uzma)

All focus group participants described prioritising their children’s needs before their own. Amongst the most socioeconomically deprived participants this involved severely reducing their own food consumption to protect the wellbeing of their children:

Jade: I won’t eat breakfast, I won’t eat dinner, I won’t eat tea, just to make sure there is enough food for the kids. Moderator: How often would that happen? Jade: Couple of times a week.

#### Looking outside the household for assistance

Family, predominantly parents and, occasionally, grandparents were identified as crucially important to survival in hard times. The apparently unconditional support available from the families of many participants stood in contrast to their experience of the world beyond the family:

To cope (with food shortages), I went to my mum’s for emotional support and for food – I would always be able to go to my mum’s.

(Sabira)

Family members provided emotional, childcare and material support, most often food; they helped avoid isolation in times of hardship; and provided skills that could be used to avoid or mitigate food insecurity, such as cooking skills. Pakistani participants were more likely to rely on extended family members, a finding partly related to housing and migration circumstances – for instance, one participant lived in a household of 13 family members, including their husband’s parents and siblings:

Most families are extended and people rely on their extended family. Like everyone in my family chips in, if one pays a bill, one does a shop.

(Basma, translated from Urdu)

However, family was not necessarily an unproblematic source of help. The ability to seek assistance from family members was, for some participants (*n*=2), precluded by inter-generational poverty while, for others (*n*=2), seeking help transgressed the ethic of independence which permeated some families. Participants who drew upon parental support in times of food insecurity either described previously assisting their parents with material resources or substituting their unpaid labour for the resources received, thereby retaining self-esteem and autonomy:

I would help out a lot at home to repay the debt. I would work really hard, I would clean and cook; it would be nothing just to make an extra chapatti – four rather than three.

(Sabira)

Mutual support systems were almost completely mediated through women, reflecting and reinforcing the gendered organisation of care within families. Most of the day-to-day help received came from other women, their mother and their partner’s mother played an especially important role in this informal economy of care, proving childcare and material support in kind rather than cash.

Child benefit was a crucial source of independent income, paid direct to the mother, which was a lifeline when other sources of income were withheld – for instance, in the case of benefit sanctions – or where their partner controlled and withheld from them other benefit payments:

It is different in England because you (the woman) gets your Child Benefits and your Tax Credits and you manage the money. He works and brings home money but you also have control.

(Sabira)

As Sabira’s comment illustrates, child benefit was a key distinguishing factor for focus group participants between the UK and Pakistan, not only providing a basic minimum income, but also endowing women with a form of autonomy and control.

### Apparent reasons for the lower prevalence – or lower reporting – of food insecurity amongst Pakistani participants

#### Social and familial networks

Well-established family networks were central to the day-to-day life of most Pakistani participants in the focus groups. Characteristically, participants lived with or very close to extended family; family members shared caring and food responsibilities within the household and provided accessible support networks. Amongst food secure Pakistani participants (*n*=7), there was no shame in sharing food and caring responsibilities or requesting assistance from extended family members – most notably in the case of women who were unable to purchase food from local shops due to language or knowledge barriers. The single food insecure Pakistani participant did not consider accessing food and financial support from her immediate family (parents) shameful, but support from extended family was not mentioned.

Food itself was commonly shared not only with family members but also with neighbours. Multiple women (*n*=5) described cooking more food than was required for household members to share with neighbours or visitors. While the gift of food was not necessarily contingent upon reciprocation, prepared food was regularly reciprocated by those who were also Pakistani/South Asian:

If you live in the heart of an Asian community food is always circulating. Neighbours give to neighbours; you cook a little extra as standard and give to others.

(Maisa)

#### Cultural and religious frameworks

The sharing of food was both culturally and religiously[2] informed. Pakistani participants explained that food was most commonly shared between neighbours during religious festivals, especially Ramadan and Eid when food was also donated to and available from mosques:

In Ramadan, I cook for four or five families to be generous. There is a particular blessing for providing food for the fasting person. It is called Iftar.

(Uzma)

Religiously informed sharing of food also operated outside religious festivals, yet this apparently cultural practice remained underpinned by religious doctrine:

It is part of Islam to give to your neighbours, even if your neighbours are non-Muslims. It is written in the Qur’an that you must give to them if you have a full stomach and they have gone hungry. But you give anyway, even when you don’t know they are hungry – you can’t ask!

(Abida)

It is said in the Qur’an that it is bad not to give food to your neighbours if someone is hungry while you are well fed.

(Hana)

However, the dialogue amongst Pakistani women in the focus group suggested that religious doctrine and practice was of less significance than the more general contribution of food to the proper functioning and maintenance of honour within Pakistani and, more generally, South Asian households. Providing food for household members and guests, in conjunction with conserving the financial security of the household, was central to the self-esteem and honour of the individual, particularly the mother, who held overall responsibility for care and food. Accordingly, the inability to provide food for family members or guests – due to financial constraints – was profoundly shameful. Indeed, this sense of shame was so acute that other items would be eschewed to ensure adequate food could be purchased:

I would rather have good food on the table than go on holiday or have flashy gadgets. Living within your means is key […] Providing adequate food is just so fundamental to South Asian families.

(Uzma)

While the shame of food insecurity was not, by any means, unique to Pakistani women, its power over both consumption and women’s openness about food insufficiency appeared to be more profound than amongst the white British women in the sample. Food insecure white British participants tended to prioritise household items and utilities, including electricity and gas bills, before food; by contrast, Pakistani women described prioritising food above all else.

#### Resource management within the household

Amongst Pakistani women, knowledge of food banks was limited and, as discussed above, some women (*n*=3) interpreted issues around food access not in terms of finance but in relation to other structural obstacles such as knowledge, language and transport. There was apparently a greater tendency amongst Pakistani than amongst white British women in the sample to cook a single meal for the entire family and eat communally. Pakistani participants described consuming a hybrid diet incorporating both traditional South Asian food, such as chapattis and dhal, and “Western” food:

My family eats chicken three times a week and we also have fish. Twice a week we eat food from outside, like Panini or fish and chips.

(Hana)

### Food aid in the context of poverty

#### Experiences of charitable food aid

A minority of participants (*n*=3) accessed charitable food aid. Those who had visited a food bank described the experience as unpleasant and undignified: processes within the food bank and condescending behaviour of staff reinforced existing inequalities between clients and food bank volunteers. The inability of clients to reciprocate the “gift” of food was considered a reason for the stigmatising conduct of staff members:

Jade: You get your voucher and you wait for the taxi to go home and they ask you to move. They say, “Can you move now please?” But as soon as people turned up with donations they were all pally. Moderator: Why? Jade: Because we are not giving food, we’re taking food from them. We’re like scum to them but when people bring food they’re happy.

The process of collecting the food parcel was described as inflexible and isolating. The indignity of receiving a food parcel was reinforced by the content of the parcel itself, which was reported to be disassociated from the needs of clients. However, despite such criticisms, food charity was appreciated and its continuation supported: it is “better they are there “cos you know that if you need, there is someone there to support you” (Danielle).

#### The absence of charitable food aid in the context of poverty

There appeared to be three reasons for the non-use of food aid: first, it was not required; second, it was avoided; and, third, the knowledge of food aid was limited or non-existent.

##### Food aid is not required

As discussed above, participants drew upon a variety of strategies to manage food shortages and thereby avoid formal charitable or state support systems. In particular, the material support provided by family members, predominantly mothers, enabled women to evade food aid:

If we did really, really struggle and his mum couldn’t help us we would go to the food bank but, like I say, his mum helps out and we always manage to get something together.

(Gemma)

##### Food aid is avoided

The humiliation and shame equated with charitable food aid was presented by participants as a key reason for its avoidance. Intense shame borne of financial insecurity and food insufficiency prevented women, even in severe food insecurity, from accessing support outside the immediate family. The perceived shame of using a food bank was not limited to the individual but also impacted the family, who would intervene before charitable food aid was sought:

There would definitely be some form of intervention before it got to the stage where someone was going to a food bank. The family would intervene and help out financially.

(Maisa)

Importantly, the impact of shame on non-use of food aid was not specific to Pakistani women, white British food insecure participants described the food bank as a last resort, avoided “nine times out of ten” or used only on condition of anonymity.

##### Knowledge of food aid is limited or non-existent

Knowledge of food aid was extremely limited amongst all Pakistani participants and apparently non-existent amongst the least affluent Pakistani women in the sample (*n*=3). While this may have been a factor in their non-use of food aid, it is more likely that food aid was not used because of the reasons presented above: shame associated with food insecurity and seeking food aid outside immediate family; robust familial/social support networks; and the apparently low prevalence of food insecurity amongst the Pakistani community in Bradford. Poor knowledge of food banks was not a factor in non-use amongst white British participants, all of whom were aware of the food bank concept and knew of their existence in Bradford.

## Discussion

### The prevalence, nature and experience of food insecurity

Food insecurity was, or had recently been, experienced by just under half of the participants. Given that participants were sampled according to their (low) socioeconomic status, this relatively high prevalence of food insecurity is unsurprising. The (small) sample size precludes meaningful comparison of food insecurity prevalence rates with emerging data showing high food insecurity amongst UK populations (FSA, 2017). Nevertheless, consistent with quantitative work by Power, Uphoff, Pickett, Stewart-Knox, Small and Doherty (2018) on the socio-demographics of food insecurity in the UK, food insecurity prevalence varied by ethnicity, albeit in a converse direction to North American research (Coleman-Jensen *et al.*, 2014): the ethnic minority group reported a lower prevalence of food insecurity than the ethnic majority. Unsurprisingly, and in line with a growing catalogue of UK research, the severity of food insecurity increased in correspondence with socioeconomic deprivation (Loopstra and Tarasuk, 2013). Children and – especially – adults living in the greatest deprivation and, concomitantly, the most severe food insecurity often experienced hunger, as identified by Harvey (2016) in her study of low-income families in London.

Graham (1993) identified two distinct but overlapping sets of strategies employed by mothers in low-income households to reconcile caring responsibilities and financial obligations. Those that sought to make ends meet within household income and those that looked to outside sources of support (Graham, 1993). Our study, conducted 23 years later, identified the same two sets of strategies. The former included cooking from scratch and in bulk; judicious budgeting; and prioritising children’s food needs over those of adults. Consistent with North American literature, women in the most socioeconomically deprived households regularly skipped meals to protect the diet of their children (Tarasuk *et al.*, 2007), at significant cost to their own physical and mental health. Indeed, there is a growing body of evidence that maternal health, amongst both white British and Pakistani women, is compromised by food insecurity (Power, Small, Doherty, Stewart-Knox and Pickett, 2018).

The latter set of strategies pivoted on emotional, material and childcare assistance from, predominantly female, family members, as identified by Graham (1984) in her work with low-income women in the 1980s. Familial assistance was fraught with complications, no least the entrenchment of gender roles in relation to care. Further complications included the impact of an individual’s “ethic of independence” on their likelihood of seeking family assistance (Graham, 1993, p. 165), the attendant perceived need to reciprocate despite poverty, as well as the impact of inter-generational poverty on the very availability of assistance (see also Burke *et al.*, 2018). In addition to social and familial support, Child Benefit was an important source of income, particularly in situations of domestic/financial abuse. A key feature of Child Benefit is that it provides the woman with an independent source of income because it is paid directly to her. This income can then be used to address food insecurity or for any other purpose she feels demands priority.

### How and why does food insecurity differ by ethnicity?

Five of eight white British but only one of eight Pakistani participants reported present or previous food insecurity. This reported near absence of food insufficiency amongst Pakistani participants conflicted with the moderate prevalence of food insecurity amongst Pakistani women identified elsewhere (Power, Uphoff, Pickett, Stewart-Knox, Small and Doherty, 2018). However, conversations with Pakistani respondents in the context of the one-to-one interview and the latter stages of focus groups, suggested that food insecurity was, indeed, experienced by Pakistani households, but concealed either from the local South Asian community itself or from charitable and state support systems.

The reported near absence of food insecurity amongst Pakistani participants could be attributed to social and familial networks, resource management within the household and cultural and religious frameworks. Pakistani participants existed within established and reliable family networks. Food was often cooked by multiple family members and for large family units, which distributed responsibility for food and limited the risk of food insecurity. Food was regularly reciprocated between neighbours, regardless of food need, possibly reducing food shortages in one or many parts of the community (see also Fitchen, 1987) and, thus, functioning as an informal social security network, yet one which was apparently unconnected to established systems of welfare provision, such as Zakat (Dean and Khan, 1997). Such reciprocation, a widespread cultural practice, was informed by Islamic doctrine and, accordingly, the sharing of food was particularly pronounced during religious festivals. Whilst white British populations may also rely on social and familial networks for informal food support, in this study, such support was less well-established and more inconstant amongst white British than amongst Pakistani participants.

As identified by Mellin-Olsen and Wandel (2005) in their study of Pakistani immigrant women in Oslo, hospitality was of importance and enjoyment, with food being a vehicle for its expression. As a consequence, food insecurity – or more specifically the inability to provide food for family members and guests – was profoundly shameful. The intense dishonour of failing to provide food spurred Pakistani women in this study to sacrifice general household items and luxuries in favour of food, and to conceal any indication of food insecurity from all but immediate family members. The limited research on food budgeting within South Asian, including British Pakistani, households suggests that most women go to considerable lengths to satisfy household food requirements, prioritising food shopping and employing a range of strategies for the optimal utilisation of food budgets (Harriss, 2008). The discrepancy between the moderate prevalence of food insecurity amongst Pakistani women identified elsewhere (Power, Uphoff, Pickett, Stewart-Knox, Small and Doherty, 2018) and both the apparent non-existence of food insecurity amongst Pakistani women in this study and their non-use of local charitable food aid (Power, Doherty, Small, Teasdale and Pickett, 2017) may be partly attributed to the perceived shame of food insecurity within the Pakistani community and a, concomitant, reticence to disclose indications of household food insufficiencies.

### Why do people use and not use food aid, and is this influenced by ethnicity?

A small minority of participants (*n*=3), and only half of those categorised as food insecure, had accessed charitable food aid. This reflects the disparity between FSA (2017) food insecurity figures and The Trussell Trust (2017) food distribution numbers, and is consistent with Canadian evidence that only a minority of food insecure individuals utilise charitable food aid (Loopstra and Tarasuk, 2015). The sample of participants using food aid is, thus, very small and the results must necessarily be interpreted with caution. Our findings echo the work of van der Horst *et al.* (2014) in Germany, Purdam *et al.* (2015) in the UK and Poppendieck (1998) in North America: the experience of receiving food from the food bank was humiliating and profoundly shameful. Staff behaved condescendingly towards clients, the content of the food parcel was disassociated from the needs of the client and the personal stigma associated with seeking food aid was acute (van der Horst *et al.*, 2014). However, as discussed by Garthwaite (2016), the continued existence of food aid was preferred to its absence and care received from some members of staff appreciated.

It is well established that a majority of people experiencing food insecurity do not access charitable food aid (Loopstra and Tarasuk, 2015); it is less clear, however, why and how these individuals avoid formal food support. In this small study, there were three reasons for the non-use of food aid: first, food aid was not required because of resource management strategies within the household and assistance from familial and social networks; second, food aid was avoided out of shame and embarrassment, with women sacrificing their own food for the wellbeing of their children; and, third, knowledge about the existence of food aid was poor or non-existent. The final reason applied to Pakistani women only. It is arguable that the white British participants, all of whom were born in the UK, could have developed better knowledge of local charitable food support than recent migrants from Pakistan. However, as only three of the eight Pakistani women in the sample were not born in the UK, it is also possible that the apparent food security of Pakistani participants and the intense sense of shame surrounding potential food insecurity were more important obstacles to accessing food aid than limited knowledge of charitable food support.

### Strengths and limitations

#### Strengths

This is the first study to address varying experiences surrounding food in the context of poverty/low-income amongst white British and Pakistani women in the UK. It presents new evidence on perceptions of food insecurity amongst Pakistani households and scrutinises why households of varying ethnic groups do not access food aid. The sample includes recent migrants from Pakistan, providing a fresh insight into language and knowledge barriers faced by young, female migrants.

#### Limitations

Capacity restrictions precluded the opportunity to formally assess participants’ food security with a food security measurement tool. Judgements about each participant’s food security were consequently based upon comments made within the focus group/interview concerning food insufficiency. It is possible that the context of the focus group dissuaded some women from disclosing food insecurity. The only disclosure of food insecurity by a Pakistani participant occurred in the one-to-one interview, intimating the possibility that food insecurity was hidden within the focus group. Further research using one-to-one interviews to discuss food insecurity would be valuable in assessing the validity of this theory.

The small sample precluded meaningful conclusions on experiences surrounding food insecurity and comparisons of food insecurity prevalence rates with other studies. Finally, the sample of those accessing charitable food aid was particularly small and, therefore, while the views and experiences of these women are interesting in their own right, they are not generalisable.

## Conclusion

Approaches to food in contexts of poverty varied between Pakistani and white British households, notably the Pakistani women who participated in the focus groups tended to prioritise food over household items more than the white British women. Both Pakistani and white British women were intimately influenced by a perceived shame of failing to provide food for family members; however, the status priorities of Pakistani women in the sample were such that they were less likely than the white British women to be placed in a position where food charity was the only option, thus avoiding a widely held contemporary shame of receiving charity.

From a policy perspective, food insecurity can only be tackled through a concerted effort to reduce poverty. In this study, welfare benefits, especially Child Benefit, were fundamental to women’s financial and domestic autonomy. The increasing inadequacy of the welfare state in the UK, a feature and consequence of the government’s austerity programme, jeopardises this autonomy. The unfreezing of Child Benefit and its uprating in line with inflation rates, both for the child element in Universal Credit and for Child Tax Credits, should be prioritised. However, such policy programmes will only be effective alongside a shift in the dominant narrative about why some people do – and some do not – use food banks. The possibility of hidden food insecurity amongst certain groups was a key finding of this paper. There is an urgent need for routine, national measurement and monitoring of household food insecurity in the UK to identify the extent of “hidden hunger” and improve targeted policy interventions, such as increasing uptake of welfare entitlements amongst South Asian populations (Prady *et al.*, 2016).

## Figures and Tables

**Table I:** Sample characteristics

Group	Name	Ethnicity^a^	Language^b^	Immigration status	Age	Children	Cohabitation/marital circumstance	Employment
1	Faiza	Pakistani	Urdu	Post-school (circa 16 years) immigrant from Pakistan	18–24	Twins (<5)	Lives with husband and children	Unemployed; husband employed
1	Abida	Pakistani	Urdu and English	Born in UK	30–36	1 child (<5)	Husband and child	Unemployed; husband employed
1	Basma	Pakistani	Urdu	Post-school immigrant from Pakistan	18–24	2 children (<5)	Lives with 13 family members	Unemployed; husband and other household members employed
1	Ghada	Pakistani	Urdu	Post-school immigrant from Pakistan	30–36	1 child (<5)	Husband and child	Unemployed; husband employed in a bank
1	Hana	Pakistani	Urdu and English	Born in UK	18–24	1 child (<5)	Husband and child	Unemployed; husband employed
1	Maisa	Pakistani	Urdu and English	Born in UK	30–36	3 children	Husband and children	Employed as a teacher; husband employed
1	Uzma	Pakistani	Urdu and English	Born in UK	24–30	2 children (<5)	Husband and children	Employed; (husband’s employment not disclosed)
2	Becky	White British	English	Born in UK	18–24	2 children (<5)	Partner and children	Unemployed; partner employed in catering
2	Danielle	White British	English	Born in UK	18–24	1 child (<5)	Children only (split from partner)	Unemployed
2	Jade	White British	English	Born in UK	30–36	8 children (11–12 weeks)	Partner and children	Unemployed; partner unemployed
2	Gail	White British	English	Born in UK	42–48	1 adult child	Single	Employed as community centre manager
3	Sabira	Pakistani/ British	English	Born in UK	18–24	3 children (<5)	Children only (divorced)	Unemployed
4	Fiona	White British	English	Born in UK	30–36	2 children (<5)	Husband and children	Employed in the NHS; husband employed
4	Emily	White British	English	Born in UK	18–24	2 children (<5)	Partner and children	Unemployed; partner employed
4	Gemma	White British	English	Born in UK	18–24	2 children (<5)	Husband and children	Unemployed; partner employed in catering
4	Kate	White British	English	Born in UK	30–36	1 child (<5)	Husband and child	Employed in community centre; (husband’s employment not disclosed)

**Notes:** ^a^Ethnicity was self-defined by the participant at the start of the focus group; ^b^language represents the language used by the participant during the focus group. In focus group 1, some participants used two languages, Urdu and English, to simultaneously converse with the moderator and other participants

## Notes

1. Includes the following categories of employment: semi-skilled and unskilled manual workers; casual or lowest grade workers, pensioners and others who depend on the welfare state for their income; and unemployed. See www.gov.uk/government/uploads/system/uploads/attachment_data/file/600091/households-below-average-income-1994-1995-2015-2016.pdf

2. All Pakistani participants described themselves as Muslim, thus, the religion in question was Islam.
